# Base‐Filling in Double‐Helical Nucleic Acids

**DOI:** 10.1002/open.202400088

**Published:** 2024-05-06

**Authors:** Mark Nana Kwame Afari, Tuomas Lönnberg

**Affiliations:** ^1^ Department of Chemistry University of Turku Henrikinkatu 2 20500 Turku Finland

**Keywords:** oligonucleotide, base pairing, base stacking, dynamic combinatorial chemistry, unnatural backbone

## Abstract

Base‐filling, *i. e*., post‐synthetic furnishing of an oligonucleotide scaffold with base moieties or their analogues, is an interesting alternative to the conventional approach of sequential coupling of building blocks (modified or otherwise). Reversible attachment of the base moieties is particularly attractive as it allows the use of dynamic combinatorial chemistry and usually leads to higher fidelity. This concept article summarizes the various backbones and coupling reactions used for base‐filling over the past fifteen years, discusses the impact of base stacking and pairing on efficiency and fidelity and highlights potential and realized applications.

## Graphical abstract

The efforts to develop backbone and nucleobase derivatives for robust base‐filling of nucleic acids, as well as the realized and potential applications of such reactions, are summarized. Covalent and non‐covalent interactions contributing to the efficiency and fidelity of base‐filling are discussed.

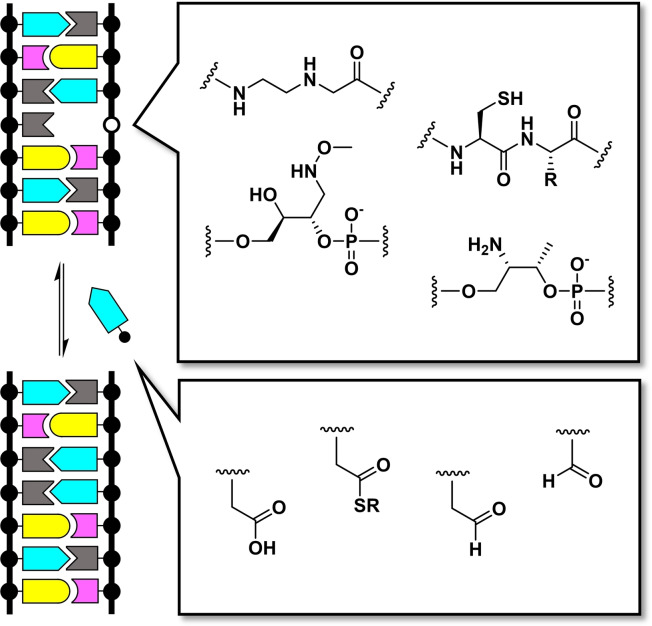

## Introduction

**Scheme 1:**
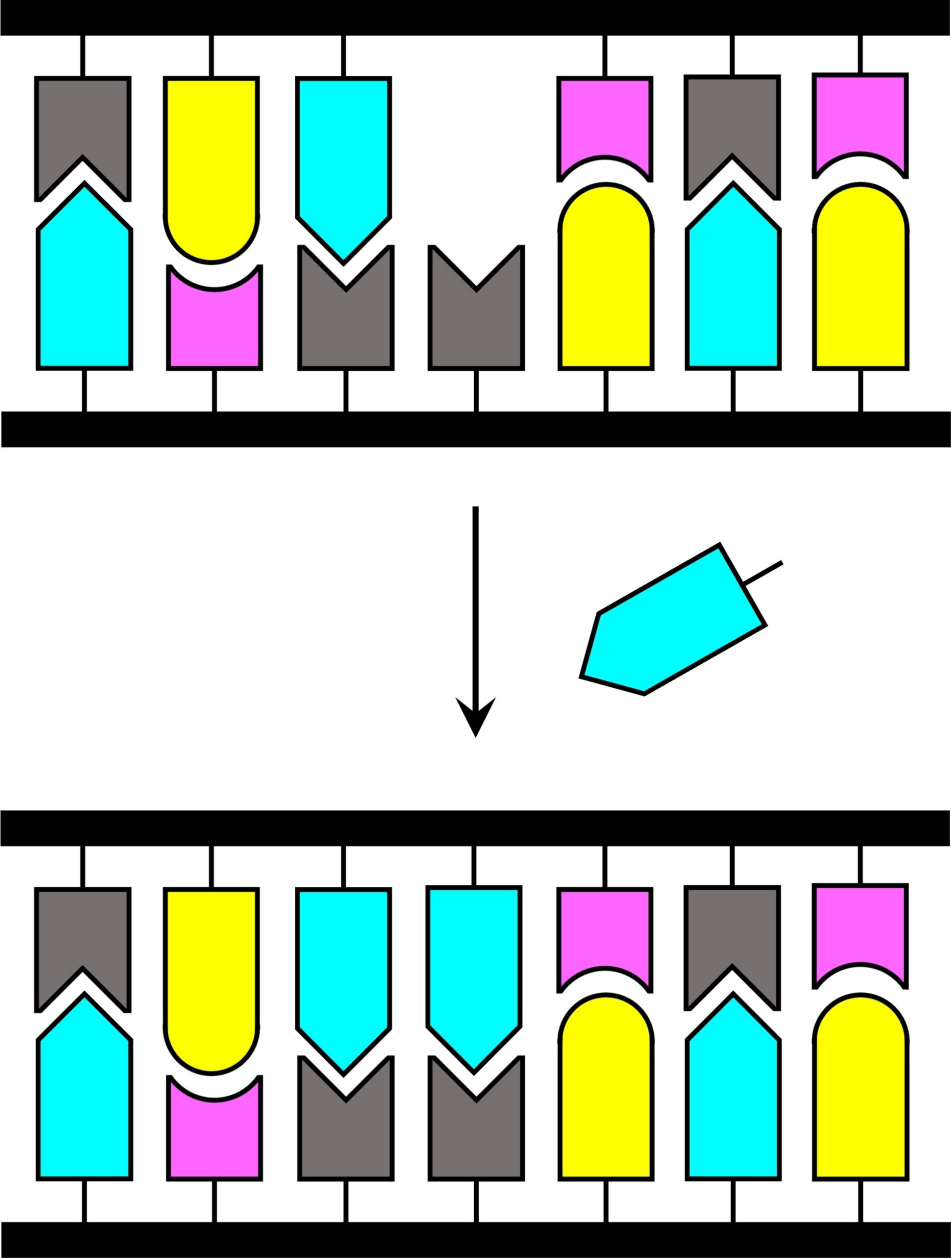
The basic principle of base‐filling of a double‐helical nucleic acid.

The concept of base‐filling was first described in 1998 by Hickman, Sreenivasachary and Lehn^[1]^ and the term coined in 2009 by Heemstra and Liu.^[2]^ Later, the term “side‐chain dynamic nucleic acids” (DyNAs) has been used for nucleic acid analogues obtained through essentially the same process.^[3]^ Base‐filling of a nucleic acid can be defined as introduction of a nucleobase (or an analogue thereof) to an abasic site on the backbone. The presence of a template strand can make this process sequence‐selective, especially if the coupling reaction is reversible (Scheme 1). The first practical examples were reported simultaneously and independently by Heemstra and Liu^[2]^ and Ura *et al*.^[4]^ in 2009. Fifteen years later, the field still remains underexplored despite the potential that base‐filling holds for various applications. This concept article discusses factors governing the efficiency and fidelity (defined as the fraction of the matched Watson−Crick base pair in the product mixture) of base‐filling, summarizes the research carried out to date and anticipates future developments.

## Backbones and Coupling Chemistries

**Scheme 2:**
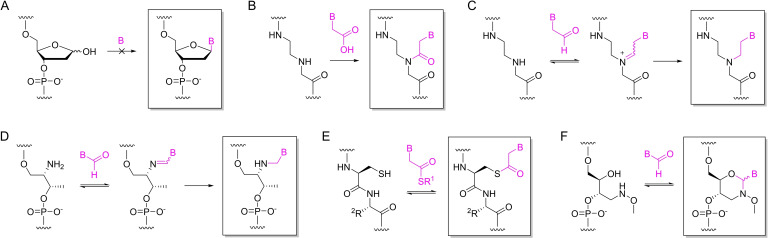
Various coupling reactions for base‐filling: A) *N*‐glycosidation (impractical under aqueous conditions); B) peptide coupling to a PNA backbone;^[2]^ C) reductive amination with a PNA backbone;^[2]^ D) reductive amination with a D‐threoninol backbone;^[5]^ E) transthioesterification with a cysteine‐containing peptide backbone;^[4]^ F) oxazinane formation with a (2*R*,3*S*)‐4‐(methoxyimino)butane‐1,2,3‐triol backbone.[^6^, ^7^] R^1^ refers to a simple alkyl group, such as methoxycarbonylmethyl, R^2^ to an amino acid side chain and B to any nucleobase.

The natural nucleic acid backbones of sugars linked to each other by phosphodiester bonds and to the base moieties by *N*‐glycosidic bonds do not lend themselves to base filling as equilibrium strongly disfavors formation of the latter in the presence of water (Scheme 2A). The peptide nucleic acid (PNA) backbone, on the other hand, is a natural choice as the peptide bond between the base moiety and a backbone amino function is readily accessible even in aqueous solution. In addition to “native” PNA, several other backbone chemistries amenable to base‐filling have been described, all featuring a strong nucleophile on the backbone and an electrophilic handle on the base moiety. For incorporation of a single base, any of the reactions discussed below can be forced to near completion by using an excess of the appropriate electrophilic derivative. Complete base‐filling of longer abasic stretches is, naturally, much more challenging and especially in the case of heterosequences still largely an unsolved problem.

PNA is an established unnatural nucleic acid backbone that hybridizes with complementary sequences with high affinity and specificity.^[8]^ It also contains a bond between the base moiety and the backbone that can be formed post‐synthetically by conventional peptide coupling (Scheme 2B). As the product is identical to the respective PNA oligonucleotide synthesized by conventional methods, it will exhibit the typical high hybridization affinity. On the other hand, the irreversibility of the coupling reaction lead to modest fidelity even though incorporation of the matching Watson−Crick base pairing partner was generally favored.^[2]^

The branching nitrogen of the PNA backbone can also undergo reductive amination with acetaldehyde derivatives of nucleobases, affording a product that is superficially isosteric with canonical PNA but has sp^3^ hybridized nitrogen and carbon atoms in place of the more rigid peptide bond (Scheme 2C). The imine formation step of this reaction is reversible, resulting in improved fidelity.[^2^, ^9^] As a tradeoff, the hybridization affinity was severely compromised on introduction of just one such residue, perhaps owing to lesser preorganization of the backbone. Indeed, replacing the achiral PNA backbone with a helically preorganized *S*‐homochiral one featuring additional side chains at the γ carbon atoms^[10]^ lead to improved hybridization affinity and selectivity.^[11]^ In addition to PNA, reductive amination has also been studied with DNA oligonucleotides incorporating a single D‐threoninol building block in the middle of the chain (Scheme 2D).^[5]^ The fidelity of base‐filling was lower than with PNA, consistent with the higher flexibility of the backbone.

The superior nucleophilicity and leaving group properties of thiols makes transthioesterification a reversible reaction in aqueous solution, at least as long as the thioester electrophiles are not trapped by other strong nucleophiles, such as amines. This reaction has been harnessed for base‐filling of peptide backbones consisting of cysteines alternating with other amino acid residues (Scheme 2E).^[4]^ Capture of thioester derivatives of nucleobases from solution could be controlled by performing the reaction in the presence of a DNA template and the resulting thioester peptide nucleic acid (tPNA) oligonucleotides exhibited sequence‐dependent hybridization with each other as well as with DNA oligonucleotides. Comparison of the fidelity of base‐filling with the results discussed above is, however, difficult as all of the experiments involved completely “naked” peptide backbones and homosequence DNA templates, rather than a single abasic site within an otherwise double‐helical oligonucleotide.

In addition to the extensively studied reductive amination, aldehydes are susceptible to another kind of reaction interesting from the point of view of dynamic combinatorial chemistry (DCC) in general and base‐filling in particular, namely formation of 1,3‐oxazolidines and ‐oxazinanes with alkoxyamino alcohols.^[12]^ Compared to reductive amination, oxazolidine or oxazinane formation bears the advantage that “freezing” of the product mixture does not require a separate reduction step, merely a shift in pH from slightly acidic to neutral. In other words, no change in hybridization of the atoms involved in the coupling reaction occurs between the dynamic selection step and the final product. The reaction between (2*R*,3*S*)‐4‐(methoxyimino)butane‐1,2,3‐triol and an aldehyde yields an *N*‐methoxy‐1,3‐oxazinane (MOANA) nucleoside analogue, with the oxazinane playing the role of the sugar ring and the aldehyde that of the base moiety (Scheme 2F).[^6^, ^7^, ^13^] Analogous to natural nucleosides (and in contrast to the structures discussed above), the base moiety is attached directly to the pseudoanomeric carbon of the sugar ring mimic, making the structure relatively rigid. So far, base‐filling of a MOANA scaffold has only been tested on aldehyde derivatives of uracil and 9‐deazaadenine. The latter was preferentially incorporated into a gap site opposite to a thymine base and the melting temperature of the resulting double helix was markedly higher than those of the other combinations tested.^[7]^

## Driving Forces and Fidelity

Base‐filling through any of the reactions discussed above appears to favor incorporation of purine, rather than pyrimidine, bases. Furthermore, positions flanked from both sides by canonical base pairs are filled much more efficiently than terminal positions.^[2]^ The most obvious interpretation for these results would be that base‐filling is mainly driven by vertical stacking interactions. With the D‐threoninol backbone, phenantroline‐2‐carbaldehyde, featuring a large stacking surface, was incorporated opposite to a cytosine residue even more efficiently than the any of the acetaldehyde derivatives of canonical nucleobases, including the Watson−Crick partner guanine.^[14]^ In this case, however, the comparison may not be entirely valid as imine formation is generally more favorable for aromatic aldehydes than their aliphatic counterparts.^[15]^

While base stacking seems to play the main role in base‐filling, base pairing is by no means insignificant. Through reductive amination on a PNA backbone, cytosine acetaldehyde was incorporated opposite to guanine 19–49‐fold more efficiently than thymine acetaldehyde and guanine acetaldehyde was incorporated opposite to cytosine 39–45‐fold more efficiently than adenine acetaldehyde, despite a similar stacking surface.[^2^, ^9^] The respective values for base‐filling opposite to adenine or thymine were somewhat lower (4–20 and 8–13), consistent with the weaker A–T base pairs. In the case of the MOANA backbone, 9‐formyl‐9‐deazaadenine exhibited a 140‐fold higher affinity for a gap site opposite to thymine than indole‐3‐carbaldehyde, a molecule with a similar stacking surface but no hydrogen bond donors or acceptors on its “Watson−Crick face”.

## Potential Applications

The first studies on base‐filling were motivated by the ambitious goal of creating lifelike dynamic combinatorial libraries of synthetic informational polymers, capable of replication and evolution.^[4]^ Compared to the more extensively studied systems based on nonenzymatic polymerization of nucleotides, such libraries would offer the advantage of avoiding the thermodynamically unfavorable esterification step. On the other hand, the primary structures of the products are intractable to conventional sequencing methods based on enzymatic polymerization. Homosequences can be fully characterized mass spectrometrically but with heterosequences one typically has to settle for base composition determined after degradation of the product. These techniques have been nicely illustrated with chimeric DNA−tPNA oligonucleotides self‐assembled on DNA templates.^[16]^ However, beyond this study, follow‐ups to the original report are scarce.

**Scheme 3:**
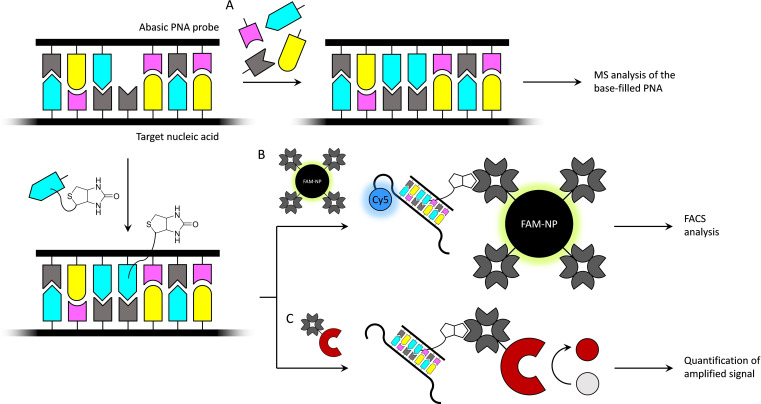
SNP genotyping by base‐filling an abasic site opposite to the polymorphic base. Identification can be based on A) mass spectrometric analysis of the product mixture of competing reactions;[^9^, ^17^] B) tethering of the target nucleic acid bearing a fluorescent tag and a nanoparticle bearing a different fluorescent tag through base‐filling and biotin−streptavidin interaction, followed by flow cytometric analysis^[18]^ or C) similar tethering of the target nucleic and a streptavidin−enzyme conjugate and quantification of the product of the enzyme‐catalyzed reaction.[^19^, ^20^, ^21^, ^22^]

A base‐filling reaction of sufficient fidelity could be used to identify a single‐nucleotide polymorphism (SNP). In contrast to established methods, only a single oligonucleotide probe is needed, placing a reactive gap site opposite to the polymorphic base on hybridization with the target sequence. This concept has been proven on PNA probes and reductive amination of nucleobase acetaldehyde derivatives, with adequate to good reliability.[^9^, ^17^] In the initial studies, characterization of the base‐filled PNA (and thus identification of the SNP) relied on mass spectrometry (Scheme 3A) but further developments have been reported more recently, based on base‐filling the PNA probe with a biotin‐functionalized nucleobase acetaldehyde, followed by immobilization on streptavidine‐coated fluorescent nanoparticles and flow cytometry (Scheme 3B)^[18]^ or signal amplification by various streptavidine−enzyme conjugates (Scheme 3C).[^19^, ^20^, ^21^, ^22^] Besides SNP genotyping, biotinylation through base‐filling and subsequent immobilization has also been exploited in PCR‐free detection and quantification of circulating microRNAs.[^23^, ^24^, ^25^]

In an approach related but opposite to the SNP genotyping discussed above, base‐filling could be used to screen for unnatural high‐affinity complements for a given nucleobase. The conventional strategy of synthesizing the corresponding protected nucleoside phosphoramidite analogues, incorporating them into oligonucleotides and finally measuring melting temperatures of the duplexes formed with the complementary strand is extremely labor‐intensive. Regardless of the coupling chemistry used, the nucleobase derivatives used in base‐filling reactions are much easier to prepare than the respective phosphoramidite building blocks. Furthermore, several derivatives can be tested in parallel in a competition assay, provided that the various products can be identified unambiguously. Pd(II) chelates for metal‐mediated base pairing with canonical nucleobases have been screened in a similar assay, although in that case the chelates were merely sandwiched between the 3′‐ and 5′‐termini of flanking oligonucleotides, rather than being covalently attached to the backbone.^[26]^

**Scheme 4:**
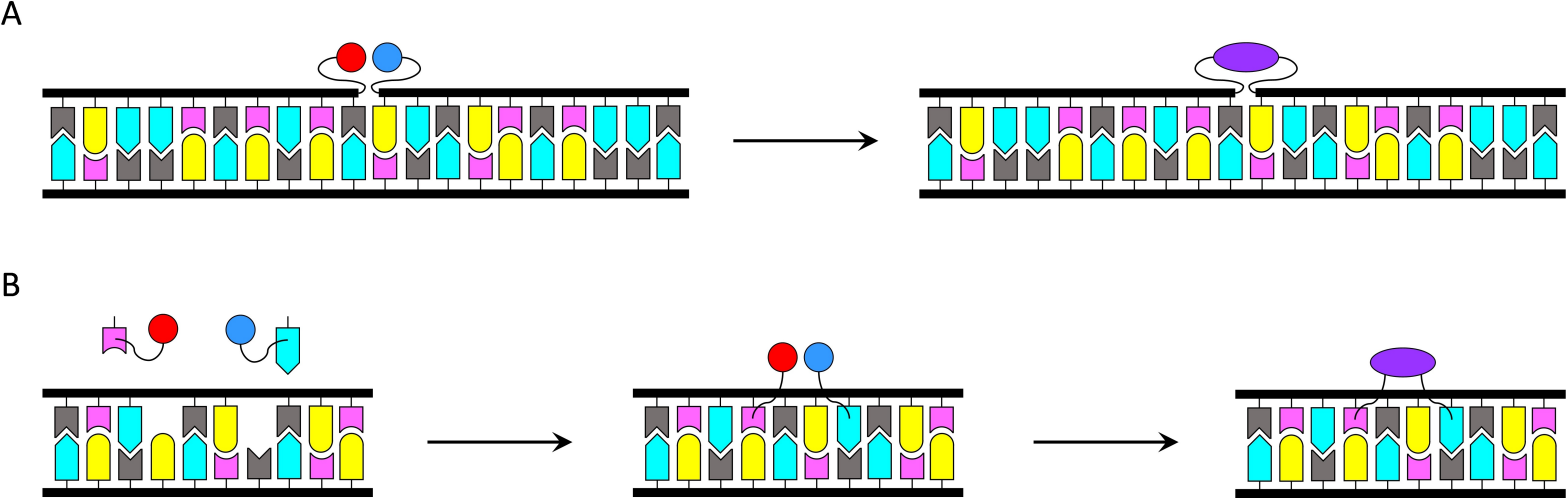
Nucleic acid templated synthesis based on either A) hybridization of the template strand with two guiding oligonucleotides, each one tethered to one reactant[^27^, ^28^, ^29^, ^30^, ^31^, ^32^, ^33^] or B) base‐filling of abasic sites within a double‐helical template by two nucleobase analogues, each one tethered to one reactant. Feasibility of the latter strategy is yet to be demonstrated in practice.

Nature uses nucleic acid templated synthesis to produce a bewildering array of different proteins from a limited pool of amino acids and the power of this approach has been harnessed also for organic synthesis in a laboratory setting.[^27^, ^28^, ^29^, ^30^, ^31^, ^32^, ^33^] In protein synthesis, aminoacyl‐transfer‐RNAs bind to the messenger‐RNA template through just three base pairs within the confinements of the catalytic core of the ribosome. Artificial examples, in turn, usually rely on DNA templates and considerably longer guiding sequences to achieve a sufficiently high local concentration of the reactants (Scheme 4A). As a result, the atom economy of such processes is far from ideal. In the case of reactions between only two reactants (representing a majority of all synthetically useful reactions), base‐filling at two abasic sites opposite to two different templating nucleobases could offer sufficient selectivity and proximity effect with a much lighter scaffold (Scheme 4B). Reversible coupling chemistries, such as oxazinane formation in the MOANA backbone,[^6^, ^7^] appear particularly attractive as they would allow easy dissociation of the product and recycling of the abasic oligonucleotide scaffold.

## Summary and Outlook

Base‐filling offers a fundamentally different approach for the synthesis of modified nucleic acids, compared with the conventional step‐wise chain assembly (enzymatically or on an oligonucleotide synthesizer), and also serves as a tool for studying the interactions of artificial nucleobase analogues within a double‐helical environment. Of the various backbone chemistries studied, base‐filling of PNA yields either the canonical structure (through peptide coupling) or a close analogue of it (through reductive amination) so it should not be surprising that so far the most promising results have been obtained on PNA. As could be expected, the highest efficiency and fidelity are observed when filling a single abasic site within an otherwise double‐helical nucleic acid and a number of useful applications based on such systems have already been described, notably in the field of SNP genotyping and detection of circulating microRNAs. Templated base‐filling of longer abasic stretches is complicated by challenges in determining the sequence of the resulting oligonucleotide but would provide a novel and highly interesting framework for artificial biology.

## Conflict of Interests

The authors declare no conflict of interest.

1

## Biographical Information

*Mark N. K. Afari earned his B.Sc. in chemistry at the University of Cape Coast, Ghana, in 2015 and his M.Sc. in organic chemistry at the University of Eastern Finland under the supervision of Professor Janne Jänis in 2017. He is currently pursuing his Ph.D. at the University of Turku, Finland, under the guidance of Professor Tuomas Lönnberg. His research focuses on new artificial nucleic acid backbone structures that would allow base‐filling and functionalization through dynamic combinatorial chemistry*.

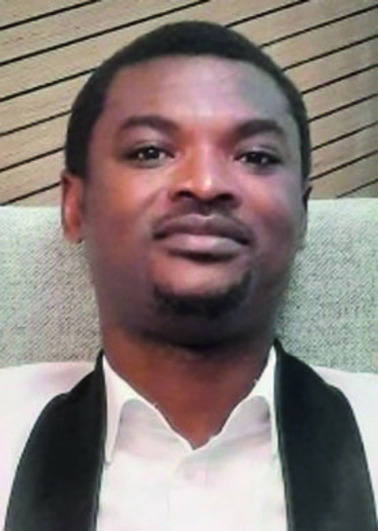

## Biographical Information

*Tuomas Lönnberg carried out doctoral studies under supervision of Dr Satu Mikkola and defended his thesis in 2005 at the University of Turku, Finland. After a two‐year term as a JSPS Post‐Doctoral Fellow in the group of Professor Makoto Komiyama at the University of Tokyo, he returned to his alma mater in 2008. In 2016, he was appointed assistant professor, in 2021 associate professor and in 2023 full professor. His fields of study include oligonucleotides bearing organometallic nucleobase surrogates, phosphate‐transfer reactions of nucleic acids and, most recently, functionalization of oligonucleotides through dynamic combinatorial chemistry*.

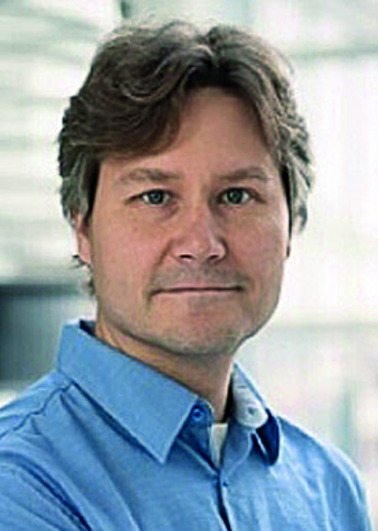

## Data Availability

Data sharing is not applicable to this article as no new data were created or analyzed in this study.
